# Prevalence and Predictors of Nonadherence to Diet and Physical Activity Recommendations among Type 2 Diabetes Patients in Southwest Ethiopia: A Cross-Sectional Study

**DOI:** 10.1155/2020/1512376

**Published:** 2020-02-28

**Authors:** Getandale Zeleke Negera, Dariowani Charles Epiphanio

**Affiliations:** Department of Clinical Pharmacy, School of Pharmacy, Institute of Health Sciences, Jimma University, Jimma, Ethiopia

## Abstract

**Background:**

Nonadherence to lifestyle modification recommendations is a major challenge in the management of diabetes mellitus. This study was conducted to measure the prevalence and predictors of nonadherence to diet and physical activity recommendations among type 2 diabetes patients (T2D).

**Methods:**

A cross-sectional study involving 322 type 2 diabetes patients was conducted from April 1 to June 30, 2019. Data were collected through face-to-face interviews using structured and pretested questionnaire. Data on sociodemographic, psychosocial, and clinical characteristics were collected. Descriptive analytical results were reported in text, tables, and figures. Logistic regression was conducted to identify predictors of nonadherence to diet and physical activity. Variables with *p* value ≤0.25 in bivariate logistic regression were considered as candidates for multivariable regression. Multivariate logistic regression was performed to identify independent predictors. Odds ratios and their 95% confidence intervals together with *p* value ≤0.25 in bivariate logistic regression were considered as candidates for multivariable regression. Multivariate logistic regression was performed to identify independent predictors. Odds ratios and their 95% confidence intervals together with

**Result:**

The rate of nonadherence to physical activity and diet was 64.3% and 36%, respectively. Female gender (AOR: 2.6, 95% CI [1.52–4.56]), age > 60 years (AOR: 2.9, 95% CI [1.12–7.42]), being illiterate (AOR: 4.2, 95% CI [1.86–9.73]), diabetes duration of >5 years (AOR: 2.5, 95% CI [1.42–4.41]), and lack of social support (AOR: 2.4, 95% CI [1.42–4.35]) were independent predictors of nonadherence to physical activity recommendations. Factors associated with nonadherence to dietary recommendations were being male (AOR = 2.8, 95% CI: [1.35–5.65]), age > 60 years (AOR = 6.3, 95% CI: [2.21–18.17]), khat chewing (AOR = 8.0, 95% CI: [3.86–16.7]), lack of social support (AOR = 15.26, 95% CI = [7.45–32.8]), and doctor's instructions or advice regarding diet (AOR = 8.9, 95% CI = [4.26–18.9]).

**Conclusion:**

The rate of nonadherence to diet and physical activity recommendations was high in the study area. Predictors of nonadherence to physical activity are female gender, age > 60 years, being illiterate, diabetes duration of >5 years, and lack of social support. Predictors of nonadherence to diet are being male, khat chewing, lack of social support, and doctor's instructions or advice regarding diet.

## 1. Background

Diabetes mellitus (DM) is a group of metabolic disorders characterized by hyperglycemia in the context of relative insulin deficiency and insulin resistance. DM could be either type I (T1D) or type II DM (T2D). Type 2 diabetes (T2D) accounts for more than 90% of the cases [1]. The number of people living with T2D has increased dramatically since the last three decades due to population growth, urbanization, increasing prevalence of obesity, and physical inactivity [2]. It was estimated that 425 million adults were living with diabetes in 2017, which is expected to rise to 629 million by 2045 [3].

Healthy lifestyle modifications such as balanced diet and physical activities are important measures in achieving optimal blood glucose control and preventing or delaying diabetes-related complications [4]. Healthy dietary habits, such as eating foods high in fibers and whole grain but low in fats, sugars, and carbohydrates, would help in decreasing the level of blood glucose and subsequently reduce the amount of insulin needed [5]. Physical activities also have a beneficial effect on glycemic control by increasing tissue sensitivity to insulin [6]. Studies showed that T2D patients who were adherent to diet and physical activity recommendations had a 40% to 60% reduction in the rate of diabetes-related complications [7, 8]. Despite the afore-mentioned benefits, the rate of nonadherence to diet and exercise recommendations ranges from 35 to 75% and 70 to 81%, respectively, among T2D patients [9–14].

Ethiopia is one of the low-income countries affected by the trend of sharp increase in the number of population with DM, where the prevalence of diabetes was reported to be 5.2% [3]. Experiences showed that most of our patients with type 2 diabetes mellitus presented with poor glycemic control which might be attributed to nonadherence to lifestyle modification recommendations. The status of nonadherence to lifestyle (diet and physical activity) recommendations and their predictors among T2D patients are not well studied in Ethiopia. Consequently, this study was designed to determine the prevalence and predictors of nonadherence to diet and physical activity recommendations among T2D patients in southwest Ethiopia.

## 2. Methods

### 2.1. Study Design and Population

A health facility-based cross-sectional study was conducted among 322 T2D patients in Jimma Medical Center (JMC) from April 1 to June 30, 2019. JMC is located in Jimma town, 355 km from the capital city Addis Ababa in the southwest, Jimma zone, Oromia regional state, Ethiopia. It is one of the oldest public hospitals in the southwest part of this country and operates under the administration of Jimma University. It is currently the only teaching and specialized hospital in the southwest region of Ethiopia. The hospital serves as a referral site and provides specialized care for southwest Ethiopia with a catchment population of 15 million.

The study population was T2D patients who attended diabetic clinic of JMC. Individuals aged ≥18 years and previously diagnosed with T2D for at least 3 months were included. Patients with the diagnosis of type 1 or gestational diabetes, acute illness, and mental impairment were excluded from study.

### 2.2. Sample Size Determination and Sampling Technique

The minimum sample size was calculated using the formula *n* = *Z*^2^pq/d^2^ (where *n* is the required sample size; *p* is the expected prevalence of nonadherence to diet and physical activity, 50% (since there is no study in this area); i.e., *q* = 1 − *p*; and *d* is error (precision), i.e., 5%). Thus, (1.96)^2^ (0.5) (0.5)/0.05^2^ = 384 patients. Since the target population is less than 10,000, we used the correction formula *nf* = No/1 + No/N, where *nf* is the corrected sample size and *N* is an estimate of population size, which was 1606. The corrected sample size was *nf* = 384/1 + 384/1606 = 308. By adding 5% contingency, the final sample size was 322 patients. A simple random sampling technique was used to select patients using a computer generated random number.

### 2.3. Study Instruments and Validation

The data collection tool was developed based on previous similar studies [15–19] and patient follow-up data. It was originally prepared in English, then translated into local languages (Afaan Oromo and Amharic), and back translated into English by independent language experts to ensure the translated version gives the proper meaning.

The questionnaire consisted of three sections: section one contained sociodemographic and behavioural characteristics; section two assessed the psychosocial and clinical characteristics; and section three included questions regarding the lifestyle modification recommendations.

For section one, the collected sociodemographic and behavioural data were age, sex, marital status, area of residence, occupation, level of education, monthly income, smoking status, and khat chewing.

Section two consisted of 3 questions regarding psychosocial and clinical characteristics, namely, family support, doctor's instructions regarding diet and exercise, and duration of diabetes.

Section three contained questions about the recommended lifestyle modifications for diabetes patients such as nonadherence to dietary recommendations, physical activity recommendations, and reasons behind nonadherence.

The questionnaire was evaluated for face validity by the team of endocrinologists and public health specialists. After considering comments of the team, a pilot study was conducted on 32 patients (10% of the sample size), based on which the final revision to the questionnaire was made for clarity and understandability.

### 2.4. Data Collection and Measurements

Data were collected through face-to-face interviews using structured and pretested questionnaire. The questionnaire was administered by 4 trained nurses and 2 supervisors. Each participant took 10–15 minutes to finish the interview. Individual interviews were conducted during the patient waiting time. Informed consent was obtained from each participant after the objectives of the study had been explained. Anonymity and confidentiality of data were assured.

In this study, the dependent variables were adherence to diet and adherence to physical activity recommendations. Healthy dietary recommendations are comprised of fruits and vegetables and foods high in fiber and whole grain but low carbohydrates (nonstarchy), fats (milk and dairy products), and sugars. Participants were considered nonadherent to dietary recommendations if they had self-reported adherence of less than three days a week (healthy diet for at least four days in the week was considered good adherence).

Physical activity recommendation includes 150 minutes or more of moderate-to-vigorous intensity aerobic activity per week or at least 30 min/day [20]. Participants were regarded as nonadherent to physical activity recommendation if he/she exercises less than 150 minutes per week.

The prevalence of nonadherence to diet and physical activity (dichotomized, yes/no) was calculated as a proportion of patients who did not adhere to the above diet and physical activity recommendations. The reasons behind the nonadherence were measured.

### 2.5. Data Quality Assurance

The quality of data was assured by properly designing the tool, the questionnaire was pretested in JMC using 16 patients, and important modifications were made prior to the actual data collection. A 2-day training was provided to the data collectors and supervisors on the data collection tool, how to ask questions, and the way of approaching respondents. The collected data were checked carefully on a daily basis for completeness, accuracy, and clarity by a supervisor, and the principal investigators monitored the overall activities of data collection.

### 2.6. Ethical Consideration

The study was approved by the Institutional Review Board (IRB) of Jimma University. The principal investigator or data collectors briefed the aim of the study to the patients, and signed informed consent was taken from all participants prior to data collection. During data collection, confidentiality was ensured and, for this reason, name and address of the patient were not recorded in the data collection checklist.

### 2.7. Statistical Analysis

Data were entered into EpiData 3.2 and exported to statistical package for social sciences (SPSS) version 21.0 software for cleaning and analysis. Descriptive analysis was performed and the results were presented by text, tables, and figures. Chi-square test was performed to check the adequacy of cells before performing logistic regression. Bivariate and multivariate logistic regressions were carried out to assess independent predictors of nonadherence to diet and physical activity. Bivariate logistic regression was performed to identify candidate variables for multivariable logistic regression. Variables with *p* value ≤0.25 in bivariate regression were considered as candidates for multivariate regression. Multivariate logistic regression was performed using backward method to identify independent predictors. Odds ratios and their 95% confidence intervals together with *p* value ≤0.05 were used to identify independent predictors of nonadherence to diet and physical activity. Goodness of fitness of the final model was checked using Hosmer and Lemeshow statistic.

## 3. Result

### 3.1. Sociodemographic, Psychosocial, and Clinical Characteristics of the Study Participants

A total of 322 (50.9% males) type 2 diabetes patients were enrolled in the study. The mean (±SD) age of the study participants was 44.2 (±1.3) where 51.2% were aged below 40 years. The sociodemographic, psychosocial, and clinical characteristics of the study population are depicted in Table 1.

### 3.2. Prevalence of Nonadherence to the Recommended Lifestyle Modifications

In this study, the prevalence of nonadherence to physical activity was 207 (64.3%) and nonadherence to diet was 116 (36%). The most common physical exercise performed by the participants was walking, 111 (96.97%), and more than half, 67 (58.26%), of them perform physical exercise twice a week (Table 2).

The most common healthy diet habit indicated by the participants was “Eating fruits and vegetables”, 162 (78.6%). About 95 (46.1%) of the patients followed healthy diet at least thrice a week (Table 3).

### 3.3. Reasons behind Nonadherence to the Recommended Lifestyle Modifications

#### 3.3.1. Reasons behind Nonadherence to Physical Activity

**Figure 1:**
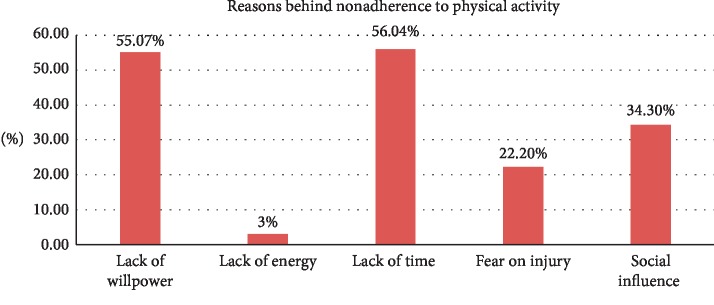
Reasons behind nonadherence to physical activity recommendation at JMC (*n* = 207).

Lack of time (56.04%) was the commonest reason for nonadherence to physical activity recommendations (Figure 1).

#### 3.3.2. Reasons behind Nonadherence to Dietary Recommendations

**Figure 2:**
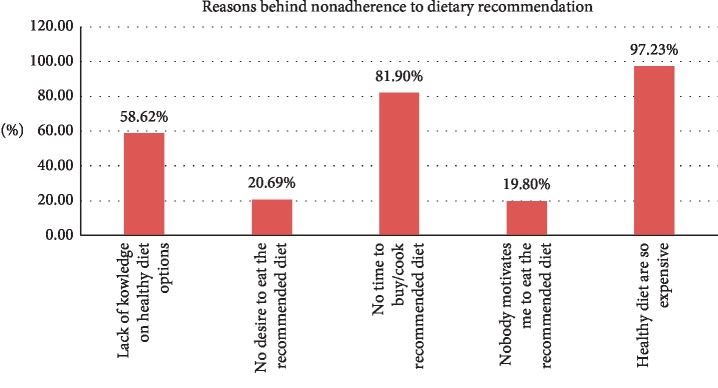
Reasons behind nonadherence to dietary recommendations at JMC (*n* = 116).

The commonest reason behind nonadherence to dietary recommendations was a belief that “Healthy diets are so expensive”, 113 (97.23%) (Figure 2).

### 3.4. Predictors of Nonadherence to the Recommended Lifestyle Modifications

#### 3.4.1. Predictors of Nonadherence to Physical Activity Recommendations

Binary logistic regression was done to identify the association between baseline sociodemographic, psychosocial, and clinical characteristics and nonadherence to physical activity recommendation. Accordingly, sex, age, residence, educational status, monthly income, smoking status, family support, and duration of the diabetes had a *p* value of <0.25.

Further multivariate logistic regression was conducted to identify independent predictors of nonadherence to physical activity. After adjusting for all variables in multivariate analysis, female gender (AOR: 2.6, 95% CI [1.52–4.56]), age >60 years (AOR: 2.9, 95% CI [1.12–7.42]), being illiterate (AOR: 4.2, 95% CI [1.86–9.73), diabetes duration of >5 years (AOR: 2.5, 95% CI [1.42–4.41]), and lack of social support (AOR: 2.4, 95% CI [1.42–4.35]) were found to be independent predictors of nonadherence to physical activity recommendations (Table 4).

#### 3.4.2. Predictors of Nonadherence to Dietary Recommendations

On a multivariate model (Table 5), independent predictors of nonadherence to dietary recommendations were male gender (AOR = 2.8, 95% CI: [1.35–5.65]), age >60 years (AOR = 6.3, 95% CI: [2.21–18.17]), khat chewing (AOR = 8.0, 95% CI: [3.86–16.7]), lack of social support (AOR = 15.26, 95% CI: [7.45–32.8]), and doctor's instructions or advice regarding diet (AOR = 8.9, 95% CI; [4.26–18.9]) (Table 5).

## 4. Discussion

This study showed that the prevalence of nonadherence to diet and physical activity recommendations was high. The commonest reason for nonadherence to physical activity was lack of time, while for dietary recommendations it was the belief that “Healthy diets are so expensive.” Gender, older age, level of education (illiteracy), khat chewing, diabetes duration of greater than 5 years, lack of doctor's instructions or advice, and social support were independent predictors of nonadherence to diet and physical activity recommendations.

In the current study, more than one-third (36.0%) and almost two-thirds (64.3%) of the patients did not adhere to diet and physical activity recommendations, respectively. In this study nonadherence to physical activity was commoner than nonadherence to diet. Nonadherence to physical activity was higher in the present study than that in Nepal [46%] [21], USA [31%] [22], Calgary [52%] [14], and Hungary [33.8%] [23] but lower than the study conducted in Yemen [84.8%] [16]. The rate of nonadherence to dietary recommendations was higher in the current study than those in USA [25.2%] [22] and Ohio [33.4%] [24] but lower than the studies conducted in Kuwait (63.5%) [25], Nepal [41%] [21], and Saudi Arabia (67.9%) [26]. This deviation might be due to a difference in socioeconomic status, education, and patient care.

### 4.1. Predictors of Nonadherence to Physical Activity Recommendations

This study also identified predictors of nonadherence to the recommended lifestyle modifications. Accordingly, females were two times more likely to be nonadherent to physical activity recommendation than their male counterparts (AOR: 2.6, 95% CI [1.52–4.56]). A similar finding was reported in a study conducted in India where females were shown to be more nonadherent than males [27]. The reason might be related to sociocultural issues in Ethiopia, where females stay at home to work most of the time. In line with other studies [28, 29], older participants (age > 60 years) were significantly associated with nonadherence to physical activity recommendation (AOR: 2.9, 95% CI [1.12–7.42]). With increasing age, there will be a decline in motor activity and the risk of comorbid disease would increase, making routine physical activity difficult. Educational level was also another predictor of nonadherence to physical activity recommendation. Nonadherence level was significantly higher in illiterates than those who attended higher education (AOR: 4.2, 95% CI [1.86–9.73]). This result is in agreement with the study conducted in Addis Ababa [30]. This could be due to low diabetes self-management behaviors and low diabetes knowledge of illiterates. The duration of diabetes was one of the predictors for nonadherence to physical activity recommendations. Participants with diabetes duration of greater than 5 years were two times more likely not to engage in physical activity than those with less than 5 years (AOR: 2.5, 95% CI [1.42–4.41]). This is consistent with the study conducted by Jadawala et al. [27], where patients with shorter duration of diabetes were more adherent to exercise. This might be explained by the fact that with an increase in the duration of disease, patients might be fed up with engaging in routine physical activity. Moreover, lack of social support was significantly associated with nonadherence to physical activity (AOR: 2.4, 95% CI [1.42–4.35), similar to the finding in Bahir Dar [31]. Social support promotes adherence by providing motivation for exercise and practical help in everyday activities.

### 4.2. Predictors of Nonadherence to Dietary Recommendations

The multivariate analysis of this study indicated that male respondents were more likely to be nonadherent to dietary recommendations (AOR = 2.8, 95% CI: [1.35–5.65]). This is in line with a Nigerian study [32] but in contradiction to a Nepalese study [33] which showed females to be more nonadherent than males. Khat chewers were also more likely to be nonadherent (AOR = 8.0, 95% CI: [3.86–16.7]. This could be explained by the reason that chewing khat is associated with loss of appetite [34] and the economic burden of buying khat may not allow them to afford healthy diets. The negative effect of khat chewing on adherence was shown by other studies [35, 36]. Social support was also significantly associated with adherence to dietary recommendations (AOR: 2.4, 95% CI [1.42–4.35]). Similar results were reported from Addis Ababa [37] and Nepal [21] which showed positive association between social support and adherence to lifestyle recommendations. Social support increases adherence by facilitating self-care activities such as buying groceries and providing motivation to cope with dietary recommendations. Moreover, nonadherence to dietary recommendations was observed significantly in those patients who had not received doctor's instruction or advice regarding diet compared with those who had exposure to dietary instructions (AOR = 8.9, 95% CI; [4.26–18.9]). Similar result was shown in a study conducted in the northeast part of Ethiopia [38].

## 5. Conclusion

The vast majority of type 2 diabetes patients in Jimma Medical Center were nonadherent to diet and physical activity recommendations. All stakeholders who are involved in the management of diabetes should be aware of the alarmingly high rate of nonadherence to lifestyle modification recommendations in Jimma Medical Center. Comprehensive education about diabetes self-management, particularly on diet and physical activity recommendations, should be tailored to individual patients with particular focus on gender, older age (>60 years), illiterates, khat chewers, longer diabetes duration, doctor's instructions or advice, and social support.

### 5.1. Limitations of the Study

Our study is not free of limitation. This study included participants from only one institution and, hence, it might not infer for other diabetic patients. Self-reported dietary history and physical activity may be subjected to recall bias which might underestimate the patients' nonadherence status. Further biases such as selection and social desirability bias may also play a role. Moreover, the nature of the cross-sectional study design does not indicate a temporal relationship or causality.

## Figures and Tables

**Table 1:** Sociodemographic, behavioural, psychosocial, and clinical characteristics of the study participants (*n* = 322).

Variables		Frequency (%)
Sex	Male	164 (50.9)
	Female	158 (49.1)

Age	<40	165 (51.2)
	40–60	109 (33.9)
	>60	48 (14.9)
	Mean ± SD	44.2 ± 1.3

Marital status	Single	69 (21.4)
	Married	181 (56.2)
	Widowed	49 (15.2)
	Divorced	23 (7.1)

Residence	Urban	178 (55.3)
	Rural	144 (44.7)

Education level	Illiterate	74 (23)
	Primary	112 (34.8)
	Secondary	81 (25.2)
	Tertiary	55 (17)

Monthly income	≤2500 ETB (86.2 USD)	130 (40.4)
	>2500 ETB (86.2 USD)	192 (59.6)

Occupational status	Unemployed	22 (6.8)
	Employed	250 (77.6)
	Pensioner	25 (7.8)
	Housewife	25 (7.8)

Smoking habit	Smoker	45 (14.0)
	Non/ex-smokers	277 (86.0)

Khat chewing	Yes	150 (46.6)
	No	172 (53.4)

Family support	Yes	160 (49.4)
	No	162 (50.6)

Doctor's instructions regarding diet and exercise	Yes	188 (58.4)
	No	134 (41.6)

Duration of diabetes	≤5	205 (63.7)
	>5	117 (36.3)

ETB: Ethiopian Birr, USD: United States Dollar.

**Table 2:** Characteristics of the physical exercise adherent group at JMC (*n* = 115).

Variable		Frequency (%)
Exercise preference	Walking	111 (96.97)
	Jogging	11 (9.55)
	Heavy weight lifting	7 (6.3)
	Other^*∗*^	17 (14.9)

Frequency of exercise	Once daily	23 (20)
	Twice weekly	67 (58.26)
	At least three times a week	25 (21.74)

^*∗*^Household chores, farming, and cattle herding.

**Table 3:** Characteristics of the diet adherent group at JMC (*n* = 206).

Variable		Frequency (%)
Diet preference	High starch and fiber diet	117 (56.8%)
	Low saturated fat and caloric intake	46 (22.3%)
	Fruits and vegetables	162 (78.6%)
	Regular alcohol intake and smoking cessation	21 (10.2%)
	Eating less sugar, carbohydrate, and fat meals	141 (68.4%)

Frequency of diet adherence	Once daily	67 (32.5%)
	Once weekly	44 (21.4%)
	At least three times a week	95 (46.1%)

**Table 4:** Crudes and adjusted odds ratio (OR) for predictors of nonadherence to physical activity recommendation in JMC (*n* = 322).

Variables		Adherence to Physical activity		^*∗*^COR [95% CI]	*p* value	^*∗*^AOR [95% CI]	*p* value
		Adherent	Nonadherent				
Sex	Female	41	117	2.3 [1.47, 3.76]	<0.001	2.6 [1.52, 4.56]	0.001^*∗*^
	Male	74	90	1	1	1	1

Age	<40	54	111	1	1	1	1
	40–60	54	55	0.49 [0.30, 0.81]	0.006	0.7 [0.38, 1.18]	0.16
	>60	7	41	2.85 [1.20, 6.76]	0.018	2.9 [1.12, 7.42]	0.028^*∗*^

Residence	Urban	52	126	1.9 [1.18, 2.98]	0.007	1.2 [0.71, 1.97]	0.51
	Rural	63	81	1	1	1	1

Education status	Illiteracy	18	56	4.1 [1.89, 8.52]	<0.001	4.2 [1.86, 9.73]	0.001^*∗*^
	Primary	32	80	3.2 [1.65, 6.32]	0.001	2.1 [0.98, 4.46]	0.056
	Secondary	34	47	1.7 [0.89, 3.57]	0.101	1.6 [0.76, 3.51]	0.21
	Tertiary	31	24	1	1	1	1

Monthly income (ETB)	≤2500 (86.2 USD)	32	98	2.3 [1.43, 3.81]	0.001	0.6 [0.35, 1.08]	0.09
	>2500 (86.2 USD)	83	109	1			

Smoking	Smoker	21	94	1.7 [0.90, 3.21	0.1	0.9 [0.41, 2.26]	0.92
	Nonsmoker	24	183	1			

Social support	Yes	71	44	1			
	No	89	118	2.14 [1.34, 3.41]	0.001	2.4 [1.42, 4.35]	0.001^*∗*^

Duration of the disease (years)	≤5	88	117	1			
	>5	27	90	2.5 [1.50, 4.18]	<0.001	2.5 [1.42, 4.41]	0.002^*∗*^

^*∗*^Statistically significant at*p* value ≤ 0.05. AOR: adjusted odds ratio, COR: crude odds ratio, CI: confidence interval, ETB: Ethiopian Birr, USD: United States Dollar.

**Table 5:** Crudes and adjusted odds ratio (OR) for predictors of nonadherence to dietary recommendations in JMC (*n* = 322).

Variables		Adherence to diet		COR [95% CI]	*p* value	AOR [95% CI]	*p* value
		Adherent	Nonadherent				
Sex	Female	119	39	1	1		
	Male	87	77	2.7 [1.68, 4.34]	<0.001	2.8 [1.35, 5.65]	0.005^*∗*^

Age category (years)	<40	54	111	1	1	1	1
	40–60	54	55	1.9 [1.14, 3.08]	0.013	2.5 [0.93, 6.95]	0.06
	>60	7	41	0.5 [0.25, 1.17]	0.11	6.3 [2.21, 18.17]	0.001^*∗*^

Residence	Urban	52	126	1.1 [0.71, 1.77]	0.61	1	1
	Rural	63	81	1	1	1	1

Monthly income (ETB)	≤2500 (86.2 USD)	82	48	1	1	1	1
	>2500 (86.2 USD)	103	89	1.8 [1.14, 2.92]	0.012	1.9 [0.98, 3.87]	0.057

Khat chewing	Yes	74	76	3.4 [2.10, 5.46]	<0.001	8.0 [3.86, 16.7]	<0.001^*∗*^
	No	132	40	1	1	1	1

Social support	Yes	136	24	1	1	1	1
	No	70	92	7.4 [4.36, 12.7]	<0.001	15.6 [7.45, 32.8]	<0.001^*∗*^

Duration of the disease (years)	≤5	125	80	1.4 [[0.88, 2.33]	0.13	1.8 [0.91, 3.65]	0.087
	>5	81	36	1	1	1	1

Doctor's instruction regarding diet	Yes	141	47	1	1	1	1
	No	65	69	3.1 [1.98, 5.11]	<0.001	8.9 [4.26, 18.9]	<0.001^*∗*^

^*∗*^Statistically significant at *p* value ≤ 0.05. AOR: adjusted odds ratio, COR: crude odds ratio, CI: confidence interval, ETB: Ethiopian Birr, USD: United States Dollar.

## Data Availability

The datasets and materials used in our study are available from the corresponding author on reasonable request.

## Abbreviations

AOR: Adjusted odds ratio
COR: Crude odds ratio
CI: Confidence interval
JMC: Jimma Medical Center
T2D: Type 2 diabetes.

## References

[1] American Diabetes Association (2014). Diagnosis and classification of diabetes mellitus. *Diabetes Care*.

[2] Wild S., Roglic G., Green A., Sicree R., King H. (2004). Global prevalence of diabetes: estimates for the year 2000 and projections for 2030. *Diabetes Care*.

[3] International Diabetes Federation (2017). *Diabetes Atlas*.

[4] Shrivastava S., Shrivastava P. S. R. J. (2013). Role of self-care in management of diabetes mellitus. *Journal of Diabetes & Metabolic Disorders*.

[5] Hamdy O., Goodyear L. J., Horton E. S. (2001). Diet and exercise in type 2 diabetes mellitus. *Endocrinology and Metabolism Clinics of North America*.

[6] Colberg S. R., Sigal R. J., Fernhall B. (2010). Exercise and type 2 diabetes: the American college of sports medicine and the American diabetes association: joint position statement executive summary. *Diabetes Care*.

[7] Lachin J. M., Walker E. A., Nathan D. M. (2002). Reduction in the incidence of type 2 diabetes with lifestyle intervention or metformin. *The New England Journal of Medicine*.

[8] Boulé N. G., Haddad E., Kenny G. P., Wells G. A., Sigal R. J. (2001). Effects of exercise on glycemic control and body mass in type 2 diabetes mellitus. *JAMA*.

[9] Ganiyu A. B., Mabuza L. H., Malete N. H. (2013). Non-adherence to diet and exercise recommendations amongst patients with type 2 diabetes mellitus attending Extension II Clinic in Botswana. *African Journal of Primary Health Care & Family Medicine*.

[10] Ary D. V., Toobert D., Wilson W., Glasgow R. E. (1986). Patient perspective on factors contributing to nonadherence to diabetes regimen. *Diabetes Care*.

[11] Nelson K. M., Reiber G., Boyko E. J. (2002). Diet and exercise among adults with type 2 diabetes: findings from the third national health and nutrition examination survey (NHANES III). *Diabetes Care*.

[12] Wanko N. S., Brazier C. W., Young-Rogers D. (2004). Exercise preferences and barriers in urban African Americans with type 2 diabetes. *The Diabetes Educator*.

[13] Garay-Sevilla M. E., Nava L. E., Malacara J. M. (1995). Adherence to treatment and social support in patients with non-insulin dependent diabetes mellitus. *Journal of Diabetes and Its Complications*.

[14] Rowley C. (1999). *Factors Influencing Treatment Adherence in Diabetes*.

[15] International Diabetes Federation (2017). *Diabetes Atlas*.

[16] Alhariri A., Daud F., Almaiman A. (2017). Factors associated with adherence to diet and exercise among type 2 diabetes patients in Hodeidah city, Yemen. *Diabetes Management*.

[17] Worku A., Abebe S. M., Wassie M. M. (2015). Dietary practice and associated factors among type 2 diabetic patients : a cross sectional hospital based study, Addis Ababa, Ethiopia. *SpringerPlus*.

[18] Thomas N., Alder E., Leese G. (2004). Barriers to physical activity in patients with diabetes. *Postgraduate Medical Journal*.

[19] Muhabuura M. B. University of Nairobi prevalence and factors associated with non adherence to diet and exercise lifestyle recommendations among type 2 diabetic patients. http://erepository.uonbi.ac.ke/bitstream/handle/11295/78017/unitid-pgdrm%20mujuni%20brian%20muhabuura%20%20%20submit(autosaved).pdf?sequence=6.

[20] American Diabetes Association (2019). *Clinical Diabetes*.

[21] Ghimire S. (2017). Barriers to diet and exercise among Nepalese type 2 diabetic patients. *International Scholarly Research Notices*.

[22] Karin M., Gayle R., Edward J. (2002). Diet and exercise among adults with type 2 diabetes, findings from the third national health and nutritional examination survey. *Diabetes Care*.

[23] Balazs H., Margit K., Peter K. (2007). Self-reported medication and lifestyle adherence in Hungarian patients with type 2 diabetes. *Pharmacy World & Science*.

[24] Wendy A., Barbara B., Terry A. (2002). Perceived recommended standards of care among adults with diabetes. *The Diabetes Educator*.

[25] Serour M., Alqhenaei H., Al-Saqabi S., Mustafa A. R., Ben-Nakhi A. (2007). Cultural factors and patients’ adherence to lifestyle measures. *The British Journal of General Practice: The Journal of the Royal College of General Practitioners*.

[26] Ataur R. K., Zaki N., Al-Abdul L. (2012). Factors contributing to non-compliance among diabetics attending primary health centers in the AI Hasa district of Saudi Arabia. *Journal of Family & Community Medicine*.

[27] Jadawala H. D., Pawar A. B., Patel P. B. (2017). Factors associated with non adherence to diet and physical activity among diabetes patients: a cross sectional study. *National Journal of Community Medicine*.

[28] Alrahbi H. (2014). Diabetes self-management (DSM) in Omani with type-2 diabetes. *International Journal of Nursing Sciences*.

[29] Mumu S. J., Saleh F., Ara F. (2015). Non-adherence to life-style modification and its factors among type 2 diabetic patients. *Indian Journal of Public Health*.

[30] Bonger Z., Solomon S., Tariku E. (2018). Adherence to diabetic self-care practices andits associated factors among patients with type 2 diabetes in Addis Ababa, Ethiopia. *Patient Preference and Adherence*.

[31] Weldegiorgis T., Abate M., Tareke M. T. (2018). Self-care practices and associated factors among diabetes patients attending the outpatient department in Bahir Dar, Northwest Ethiopia. *BMC Research Notes*.

[32] Adisa R., Alutundu M. B., Fakeye T. O. (2009). Factors contributing to nonadherence to oral hypoglycemic medications among ambulatory type 2 diabetes patients in Southwestern Nigeria. *Pharmacy Practice (Internet)*.

[33] Parajuli J., Saleh F., Thapa N. (2014). Factors associated with nonadherence to diet and physical activity among nepalese type 2 diabetes patients; a cross sectional study. *BMC Research Notes*.

[34] Wabe N., Mohammed M. (2012). What science says about khat (Catha edulis Forsk)? Overview of chemistry, toxicology and pharmacology. *Journal of Experimental and Integrative Medicine*.

[35] Tareke M., Tesfaye S., Amare D., Belete T., Abate A. (2018). Antipsychotic medication non-adherence among schizophrenia patients in Central Ethiopia. *South African Journal of Psychiatry*.

[36] Soboka M., Tesfaye M. (2015). Khat use in people living with HIV: a facility-based cross-sectional survey from South West Ethiopia. *BMC Psychiatry*.

[37] Mamo M., Demissie M. (2016). Self Care practice and its associated factors among diabetes patients in Addis Ababa public hospitals, cross sectional study. *Diabetes Cholesterol Metabolism*.

[38] Ayele A. A., Emiru Y. K., Tiruneh S. A. (2018). Level of adherence to dietary recommendations and barriers among type 2 diabetic patients: a cross-sectional study in an Ethiopian hospital. *Clinical Diabetes and Endocrinology*.

